# The Promise and Challenges of Determining Recombinant Bovine Growth Hormone in Milk

**DOI:** 10.3390/foods11030274

**Published:** 2022-01-20

**Authors:** Axel Raux, Emmanuelle Bichon, Alessandro Benedetto, Marzia Pezzolato, Elena Bozzetta, Bruno Le Bizec, Gaud Dervilly

**Affiliations:** 1 Oniris, INRAE, LABERCA, 44300 Nantes, France; axel.raux@oniris-nantes.fr (A.R.); emmanuelle.bichon@oniris-nantes.fr (E.B.); bruno.lebizec@oniris-nantes.fr (B.L.B.); 2 Istituto Zooprofilattico Sperimentale Del Piemonte, Liguria e Valle D’Aosta, Via Bologna 148, 10154 Torino, Italy; alessandro.benedetto@izsto.it (A.B.); marzia.pezzolato@izsto.it (M.P.); elena.bozzetta@izsto.it (E.B.)

**Keywords:** recombinant somatotropin, milk, analytical methods, chemical food safety, public health, confirmation, screening, recombinant bovine somatotropine (rbST), rbGH

## Abstract

Recombinant bovine growth hormone (rbGH) is produced in large quantities and widely used in a number of countries worldwide to stimulate milk production in dairy animals. The use of this compound in animal production is strictly regulated by food safety directives in force, in particular in the European Union (EU). Although analytical strategies for the detection of rbGH in blood have been successfully reported over the past 15 years, they do not fully answer the expectations of either competent authorities or industrials that would expect measuring its occurrence directly in the milk. As a matrix of excretion but also of consumption, milk appears indeed as the matrix of choice for detecting the use of rbGH in dairy animals. It also allows large volumes to be collected without presenting an invasive character for the animal. However, rbGH detection in milk presents several challenges, mainly related to the sensitivity required for its detection in a complex biological matrix. This review article presents the specific difficulties associated with milk and provides an overview of the analytical strategies reported in the literature and whether they concern indirect or direct approaches to the detection of rbGH administration to animals, with applications either for screening or confirmation purposes.

## 1. Introduction

### 1.1. Historical Background

The ability of bovine growth hormone (bGH), or bovine somatotropin (bST), to temporary increase lactation yield was discovered in the 1930s when it was first extracted from pituitary gland and injected to dairy cows [1]. Later, during World War II, the galactopoietic effect of anterior pituitary growth hormone was confirmed, and it was consequently used to increase milk production [2]. However, the amount of endogenous growth hormone recovered this way was very low, and a large number of slaughtered animals was necessary to obtain the desired galactopoietic effects (200 bovines required to treat one cow for a single day) [2,3,4]. Later, multiple studies confirmed these effects and demonstrated an associated increased milk yield by 6 to 35% [5,6,7,8]. In the 1980s, thanks to the development of DNA technology, it was made possible to provide recombinant bovine growth hormone (rbGH) in large quantities. Studies around its effects and commercial applications flourished [9]. South Africa was the first country to approve rbGH in 1988, and the USA followed with an authorization given by the Food and Drug Administration (FDA) in 1994. Since then, rbGH has been commonly used in a number of countries.

### 1.2. Chemical Structure and Metabolism

Bovine growth hormone is a protein naturally produced by the pituitary gland of cattle. It is expressed into four different variants (Table 1) composed of 190 or 191 amino acids, corresponding to a 22 kDa molecular weight (Mw). Recombinant bovine growth hormone is synthetically produced by different pharmaceutical companies and for which two major forms are commercially available. Posilac was developed by Monsanto and is now commercialized by Elanco since 2008; it is the most commonly used formulation showing a difference from the endogenous form (main variant) with the alanine in the NH_2_ terminal position substituted with a methionine (Met-rbGH). Boostin is produced by Lg Chem, and its amino acid sequence is similar to that of the main endogenous variant (Ala-rbGH). The isoelectric points (pI) of bGH/rbGH have been determined with two-dimension electrophoresis and are in the range 7.8–8.2 [10].

By a succession of hormones secretions and feedbacks, growth hormone concentration is regulated in blood and tissues. Growth hormone binds to spotlight tissues cell receptors, which drives the direct action of the hormone. The indirect action is commanded by Insulin-like growth factor I (IGF-I) production, preceded by growth hormone fixation on liver cells receptor. Physiological effects in mammary tissue include increased uptake of nutrients used for milk synthesis, increased activity in the secretory cells, and increased milk synthesis. The increased mammary uptake of nutrients used for milk synthesis is accompanied by an alteration of the metabolism in other tissues, which results in the increasing availability of these nutrients for milk synthesis. In adipose tissue, a decreased lipogenesis results in leaner muscle. On the basis of these effects, bovine somatotropin is used to increase milk production in dairy cows (+4–6 kg/day) and has been shown to improve carcass composition in finishing beef steers.

### 1.3. Issues Associated with the Use of rbGH

The use of bovine growth hormone has been associated since the early 1980s with two main issues related to animal welfare and human health. 

As far as animal welfare is concerned, the available literature is mixed on the subject. Some studies in particular report significant adverse effects (mastitis, lameness, and fertility-related problems) [12], while others conclude the opposite [13,14,15,16,17,18,19,20].

With regard to human health, scientific opinions worldwide are also divided on the subject. FDA was among the first authorities to approve rbGH considering that milk and meat from cows treated with it is safe for humans to eat since the hormone is a large protein that is degraded by digestive enzymes in the gastrointestinal tract, leading to proteolytic fragments with no biological activity. Joint FAO/WHO Expert Committee on Food Additives (JECFA), in its latest assessment (2014) [21] based on a systematic review of the literature published since its previous evaluation, also concluded that there was a negligible risk to the consumer both from the point of view of the possible presence of rbGH residues (“*there is no significant change in the concentrations of total bST detected in milk and tissues of rbST-treated cows when compared with untreated controls*”) and of the possible increase in IGF-I content associated with such treatment of dairy cattle. It also concluded that there was no evidence to suggest that the use of rbGH would result in a higher risk to human health due to the possible increased use of antimicrobial agents to treat mastitis or the increased potential for non-compliant antimicrobials residues in milk. Further, JECFA could not establish a specific link between the use of rbGH and the development of antimicrobial resistance (AMR). Consequently, the Committee reaffirmed its previous decision on Acceptable Daily Intake (ADIs) as “not specified.” Following this opinion, at the European level, an European Food Safety Authority (EFSA) opinion has been issued, concluding that an increase of AMR in humans following to the use of rbGH in dairy cattle is plausible [22]. EFSA therefore established that current knowledge does not allow concluding on the absence of risk and recommend for additional investigations.

### 1.4. Regulation

Although JECFA reaffirmed its previous decision on Maximum Residue Limit (MRLs) as “not specified” for somatotropin as established since 1993 [23], there are still worldwide divergences regarding implemented risk management. In the 20 countries where rbGH employment has been approved (USA, Brazil, Chile, Columbia, Costa Rica, Ecuador, Egypt, El Salvador, Guatemala, Honduras, Kenya, Lebanon, Mexico, Pakistan, Panama, Peru, South Africa, South Korea, Venezuela, and Zimbabwe), suppliers recommend using a prolonged-release injectable formulation in single-dose syringe containing 500 mg sometribove zinc, injected every 14 days subcutaneously in the postscapular region or ischiorectal fossa, beginning during the 9th week after calving until the end of lactation. In the 27 European Member States, rbGH is non authorized according to Decision 1999/879/European Commission (EC) stating that “the placing on the EU market of bovine somatotrophin for the purpose of its marketing and use in the treatment of dairy cows by any means whatsoever,” alleging animal welfare according to EU’s Scientific Committee on Animal Health and Animal Welfare [24]. Further, the new regulatory scheme induced by the application of the Official Control Regulation [25] and repealing Dir 96/23/EC [26] confirms this provision by defining “*A substances*” as “*Prohibited or unauthorised pharmacologically active substances which may be used for illegal treatment in food producing animals*” listing “*A3 substances*” as “*Pharmacologically active substances, not listed in* Table 1 *of the Annex to Regulation (EU) No 37/2010 or substances not authorised for use in feed for food-producing animals in the EU according to Regulation (EU) No 1831/2003*”. In particular, growth hormone is from now on included under the A3e group “*Protein and peptide hormones.*” Thus, Europe firmly reaffirms its position with regard to these substances in livestock and, with a public health perspective, its commitment to the performance of the associated controls. Canada, Australia, New Zealand, Japan, and Israel have also banned the use of rbGH. In those countries where rbGH is banned, controls are performed to track down growth hormone abuse in livestock, especially in dairy cows. The effectiveness of these controls relies on the implementation of appropriate analytical strategies as described below. This implies and drives for continuous efforts to increase their performance by integrating available technological innovations that allow for lower detection limits in relevant animal matrices.

### 1.5. Analytical Strategies to Detect rbGH Abuse in Animals

The detection of recombinant bovine growth hormone administration in serum or plasma has been previously described in a number of articles as reviewed in [27,28]. In order to enlighten the reader on the different strategies available and in particular to complement the 2014 review [28] with the work published since then on the subject, an up-dated overview of these methods is presented in Tables S1 and S2, also including data on other animal species when available.

Although analytical strategies for the detection of rbGH in blood have been successfully reported, they do not fully answer the expectations of either competent authorities or industrials that would expect measuring its occurrence directly in the milk. As a matrix of excretion but also of consumption, milk appears indeed as the matrix of choice for detecting the use of rbGH in dairy animals. It also allows large volumes to be collected without invasive sampling procedures on the animals. However, rbGH detection in milk present several challenges, mainly related to the sensitivity required for its detection. Indeed, while physiological concentration of rbGH in blood is reported to range from 1 to 10 ng/mL [29,30], it is expected to fluctuate around 1 ng/mL in milk [31]. In addition, rbGH kinetic of elimination in blood suggests that the hormone is detectable only between 4 h and four days after treatment in cow serum [32]. Finally, milk is a complex matrix comprising a large number of heterogeneous proteins together with lipids, which can interfere with rbGH signals or form complexes modifying growth hormone properties and structure [33]. At a time when European regulations on the control of residues of veterinary substances are changing, and protein hormones are even more formally included in the list of substances of interest [25], it was time to take stock of the milk related strategies, their performance, and their limitations.

## 2. Specificity of Determining Recombinant Bovine Growth Hormone Administration in Milk

### 2.1. Milk Composition

Milk is a complex biological fluid whose composition varies according to the mammalian specie. In bovine, the secretion of milk starts after calving and lasts for around 10 months (305 days). This period is called lactation and is divided into three parts: early, mid, and late lactation. 

Milk is widely recognized for its high nutritive value since it contains most of the key components for the growth of the new-born, and it is a rightful energy supplier for human adults as well. It is a complex biofluid, mainly composed of water (87%) and macro-nutrients, such as lactose (4.6%), lipids (4.2%), proteins (3.4%), and micronutrients (0.9%) [34]. However, these proportions are not permanent but fluctuate under several parameters, such as specie, individual, stage of lactation, season, animal feed and health, and soil contamination [35,36]. 

Extracting and purifying a specific protein like rbGH from such a complex environment requires an accurate knowledge of these components in order to define the appropriate physical-chemical conditions and parameters for a suitable and effective protocol. Based on the physico-chemical properties of rbGH described above (Mw, pI) and considering the very low levels of concentration expected in milk (1–2 mg/mL) [31,37], the main components that should be a problem for the efficient analysis of these residues in milk are detailed below.

#### 2.1.1. Small Components

Lactose is the main carbohydrate in milk [36]. It represents around 35.3% of bovine milk dry matter mass. Minerals (Ca, Mg, Na, Fe, Zn) and vitamins (D, A, B, C, E) are present in small amount (about, respectively, 0.8% and 0.1% of total bovine milk mass). Lactose, like minerals and vitamins, is a small molecule that is easily eliminated from the matrix and should not interfere during rbGH extraction.

#### 2.1.2. Lipids

Lipids are present in milk in more than 400 different forms of fatty acids and represent around 32% of bovine milk dry matter mass. Lipids detected in milk are mainly triacylglycerols (TAGs), together with others in a smaller quantity, such as diacylglycerol, cholesterol, phospholipids, or free fatty acids [38]. Due to their physico-chemical properties, these lipid compounds can be troublesome in the rbGH extraction process. Indeed, since rbGH is hydrophobic, it could be adsorbed on lipids, leading to a loss during skimming, which is a step often described as the very first one applied when preparing the milk samples (e.g., 3000× *g*, 10 min, 4 °C) [39]. Further, the high fat content of milk is known to block and interfere the antigen-antibody binding, which may be applied as an enrichment step during rbGH extraction, leading to decreased extraction performances [39].

#### 2.1.3. Proteins

The milk nutritional value and properties are mainly related to the protein composition of milk, which exerts biological and physiological activities essential to the calf [40]. Milk protein specific composition also differs according to lactation season, stage of lactation [41], and feeding and health [42] but mainly from genetic heritage [43,44] and from a mammalian species to another [45,46].

Milk proteins are divided into two categories: the high-abundance proteins, counting for about 90% of the total protein fraction, and the low-abundance proteins. In order to have a distinction between these two fractions, a centrifugation can be performed. Milk fat globule membrane (MFGM) is located in the upper fat layer; right below, there are the high-abundance proteins; and finally, a wide quantity of proteins can be found in the cellular pellet [47].

The high-abundance protein fraction is composed of caseins and whey proteins. The ratio of caseins/whey proteins is quite different according to the species and stage of lactation, as different nutrients are needed according to mammalian species or infant growth step. In human milk, the quantity of casein increases from 10 to 50% through lactation stages [45], whereas in bovine milk, the ratio is around 80/20 during the same stages [41]. Caseins (CN) are milk phosphoproteins weighing for about 80% of the total protein fraction in bovine milk. There are different casein structures diverging from genetic variant and phosphorylation degree (Table 2). Still, four main casein types are generally described: αs1-CN (around 43% of total casein fraction), αs2-CN (11%), β-CN (34%), and κ-CN (12%). Caseins exhibit a molecular weight (Mw) around 23 KDa and an isoelectric point (pI) of 4.6. The very close Mw between caseins and rbGH already suggests a difficulty in eliminating this major protein fraction. In milk, caseins are organized as micelles, colloidal complexes composed of proteins, and calcium, whose role is to provide nutrition, increase the fluidity of individual casein proteins, and solubilize phosphate and calcium [48]. For all these reasons, caseins are supposed to be the main interfering compounds in milk when developing a method to extract and purify rbGH. 

Soluble proteins at pH = 4.6 are called whey proteins. The two major whey proteins of bovine milk are α-Lactalbumin (α-La) and β-Lactoglobulin (β-Lg), and both present in different genetic variants and accounting for, respectively, 3.6% and 9.8% of bovine milk total protein fraction. The fact that these proteins exhibit different physico-chemical properties from those of rbGH, namely a pI of 4.5 and Mw of 14 kDa for lactalbumins and pI 5.1 and Mw 18 kDa for lactoglobulins, does not make them problematic interferents in the case of rbGH analysis [10]. Other whey proteins include immunoglobulin (Ig), bovine serum albumin (BSA), lactoferrin (Lf), and lactoperoxidase (Lp), which do not appear problematic either in the present context. 

The final 2% of bovine milk protein is located in the milk fat globule membrane. Milk fat globule membrane is composed of 30% lipids (mostly phospholipids, cerebrosides, and cholesterol) and 70% proteins (among which are mucin 1, butyrophilin, and adipophilin) [38,51]. Whey proteins and Milk fat globule membrane (MFGM) proteins are not considered as potential challenge, as they are present in a relatively small amount and because their physico-chemical properties are different from rbGH ones. 

While methods for extracting rbGH from serum or commercial syringes usually involve a precipitation step with ammonium sulphate ((NH_4_)_2_SO_4_) [52], it can be anticipated that applying this strategy to milk would lead to the co-precipitation of the other proteins, in particular caseins, which are in the majority and will consequently carry the rbGH in their network, preventing its further extraction. Consequently, to deal with this protein problem, two options are possible: either the specific elimination of caseins by precipitation at their pI 4.6 before precipitating rbGH at its own pI (7.8) [10,53] or, in order to avoid these successive and sensitive precipitation steps, to extract rbGH directly from the milk, for example, by immunoaffinity, without trying to eliminate the interfering proteins [54].

### 2.2. Analytical Strategies in Milk to Detect rbGH Administration

If the strategies to detect rbGH administration in milk are the same as those applied in the blood, i.e., detection of indirect markers or detection of residues of the substance itself, the challenges are exacerbated. Indeed, the expected concentrations are at the ppb (µg/L) level [31,37], and milk is a biological matrix even more complex than blood. The following paragraphs provide an overview of the strategies developed so far and an assessment of their performance and applicability.

#### 2.2.1. Indirect Analytical Strategies

As in serum, it is possible to detect rbGH misuse with indirect methods involving the monitoring of effect markers (Table 3). Such screening methods rely on the determination of an endogenous compound, which implies setting a suspicion threshold (i.e., a level of biomarker concentration above or below which this value is not considered physiologically natural) to determine the abuse, and cannot be used as confirmatory method because it does not allow evidencing the residue itself as regulatory required according to Reg EU/2021/808 (article 2, definition 12) [55].

IGF-1 has also been reported in milk as suitable biomarker of rbGH. Some studies report its measurement with immunoassays allowing the biomarker quantification from 0.5 to 1 ng/mL using ELISA test and up to 4 ng/mL using a biosensor immunoassay (BIA), in cow’s milk [56]. 

The determination of antibodies raised against rbGH as a consequence of its administration has also been reported in milk. It generally involves flow cytometric immunoassay (FCIA) and is expected to allow the classification of rbGH-treated and -untreated animals. Although the reported performances in terms of false-negative (false compliant) rate (33%) does not comply screening performances requirements set at 5% initially in Decision 2002/657/EC and currently in Reg EU/2021/808 (§1.1.2) [55], the strategy proposes a detection window until two weeks after the last rbGH treatment, where a positive antibody biomarker response could be observed in 63% of the cows [57]. The antibody responses appear specific to rbGH and equivalent to serum responses [58,59]. A recent study reports the formation of rbST-induced antibodies in animals treated with either Ala-rbGH or Met-rbGH [37]. It was observed in both treatments that the rbGH-induced antibodies were transferred from blood to milk, showing no blood-milk barrier specificity for these antibodies. These immunoassay procedures in milk show great promises for enforcement purposes as screening tool for rbGH misuse, particularly at the tank level, which is more adequate than sampling from individual cows because not all animals show an immune response upon treatment [57].

In parallel to these approaches, a more recent alternative aiming to search for biomarkers of effects linked to rbGH administration has been proposed using a transcriptomics approach. Among other matrices (blood, hair follicles), Lamas et al. investigated milk somatic cells and 15 gene expression profiles, using real-time polymerase chain reaction (RT-PCR) [60]. Their results showed that four genes could be used as potential biomarkers in milk somatic cells: cyclin D1 (CCND1), interleukin 1 beta (IL-1β), tumour necrosis factor (TNF), and insulin-like growth factor 1 receptor (IGF-1R). In a further research, they explored 18 gene expression profiles in milk somatic cells, confirming these four biomarkers [61]. This clearly demonstrates the potential of omics strategies in the present context as was already previously demonstrated in blood and urine of rGH-treated animals [39,62,63,64].

#### 2.2.2. Direct Analytical Strategies 

A few studies report total bGH (native and recombinant forms) analytical strategies to identify rbGH treatment generally involving radioimmunoassay (RIA). An associated lower limit of detection (LOD) of 0.5 ng/mL is reported [66]. This strategy was successfully implemented by Zhao et al. to determine the effects of rbGH administration on total bGH concentration in milk, concluding an observed increased concentration in treated animals by about 60% with usual treatment conditions [67]. Then, Zwickl et al. developed an enzyme-linked immune-sorbent assay (ELISA) method that allowed, in four hours, the determination of total bGH in milk, with a LOD of 0.2 ng/mL [68]. In another study, bGH was successfully measured with an ECLIA (ElectroChemiLuminescence Immunoassay) allowing a LOD of 5 pg/mL [31]. Again, these immunoassays are powerful as screening tool yet are not usable as confirmatory methods, as differentiation between bGH and rbGH is not possible.

As of now, only mass spectrometric methods allow an unambiguous identification of rbGH from bGH providing their amino acid sequence differs, which is the case when Posilac (Met-GH) is used (Table 4). In 2010, a first attempt to transpose a serum analytical strategy to milk was reported [69]. Although this strategy allowed reaching low concentration levels with a reported decision limit (CCα) of 1.2 ng/mL for the selective identification of rbGH and its unambiguous discrimination from the native bGH, the routine application of this protocol faced robustness concerns in terms of sensitivity (spiking level 100 ng/mL), while precision (Relative Standard Deviation (RSD) 18%), trueness (RSD 7.2%), and uncertainty (u = 19.4%, U = 38.8%) measurements were found acceptable but requiring further developments. In 2012–2013, the first attempt to replace the protein precipitation step with a selective extraction step using specifically developed antibodies grafted onto magnetic beads was tested in the European Unique-Check project [70]. Although promising results were obtained as a proof of concept study, the problems related to fat interactions could not be fully solved. In 2019, Welsh et al. presented results of bGH measurements in incurred milk samples without, however, describing the protocol used and its performances [71]. More recently, a liquid chromatography–tandem mass spectrometry (LC-MS/MS) method, involving a selective antibodies purification step, describes as performance a decision limit (CCα) of 2.3 ng/mL in milk [37]. This level of performance allowed the identification of Ala-rbGH in the milk of treated animals as early as day one after administration at a level of 11 mg/mL and to continue this detection above the CCα for six days. Interestingly, the results show that the administered Ala-rbGH is transferred from blood to milk, but this is not the case for Met-rbGH, suggesting a blood-milk barrier-related specificity for these compounds. This result should now be confirmed as it may lead to a reflection on the relevance of considering milk as a matrix of choice for control, especially due to the widespread use of the Met-GH form. On a strict, analytical point of view, the future implementation of this approach proposed by the European Reference Laboratory (EU-RL) in charge of the control of growth promoters in Europe should confirm the relevance and robustness of an expected routine use, according to new EU regulatory requirements [55].

## 3. Prospects for Efficient Analysis of rbGH in Milk

While MS monitoring allows unambiguous identification of rbGH residues enabling their selective analysis, the current bottleneck of the strategy preventing its robust enforcement lies in the upstream stages of the protocol, particularly in terms of optimal extraction and purification, which are related to sensitivity and reproducibility issues. The prospects for improved performance are therefore to be sought in the preparation of milk samples.

Recent milk treatment strategies would benefit from being tested for the problem of rbGH analysis. Specifically focusing on the main interfering compounds, caseins are usually eliminated by applying a simple acidic precipitation of caseins, which in the particular case of rbGH, also induces its precipitation. This justifies that such strategy cannot be retained in the present case. As an alternative, a study reported the use of a phosphate buffer (1M; pH 6.0) to eliminate most of the caseins instead of using hydrochloric acid in the objective of extracting recombinant human factor IX (rhFIX) and recombinant hirudin (rH) in milk, which allowed a whey protein fraction containing more than 90% recovery for both recombinant hormones [72]. This approach could be tested to overcome the challenge associated to the extraction of rbGH from the micellar network formed by caseins. Over the last past years also, sample preparation strategies based on casein micelles dissociation by mixing skimmed milk with a solution composed of Bis-Tris, dithiothrénol, trisodium citrate and urea [72,73], or guanidine chloride [74,75] have been described for increased efficiency in milk protein analysis, which would be another option to avoid the casein issue in the objective of rbGH analysis.

Another strategy would be rather than eliminate interfering proteins to selectively extract the rbGH of interest. This involves the use of an approach involving structural recognition phenomena as made possible by immuno-recognition. Such a strategy has already been efficiently applied in bovine serum [76] and recently described in milk [37]. Further, on-line tryptic digestion should be considered to reinforce robustness of this step, which suffers reproducibility issues and would also offer a higher throughput in the analysis.

If the main challenges are at the level of sample preparation, the question of rbGH detection also deserves to be asked. Indeed, to date, it is mainly strategies involving residue monitoring by low-resolution tandem mass spectrometry (LRMS) that have been reported, which is in line with a recent review reporting targeted LRMS approaches as the common (>80%) analytical choice for food contaminant detection [77]. Such approaches rely on monitoring at least two MS/MS transitions in Selected Reaction Monitoring (SRM) mode for identification purposes, which may constitute a drawback. As the more analytes that are included in the method, the more necessary ion transitions have to be measured. Therefore, there is an increased chance of common or overlapped transitions affecting the method LODs. To counter this problem, High Resolution Mass Spectrometry (HRMS) targeted methods may be proposed as an alternative [78], as was probably the case in the study involving an Orbitrap for rbGH determination although not specified [71]. HRMS capabilities could also be utilized in non-targeted workflows using MS^2^ spectral libraries for suspect screening purposes. In such approaches, the MS/MS data are collected using either data-dependent acquisition (DDA) or data-independent acquisition (DIA). Such an approach that aims at capturing a broader range of compounds has recently been successfully reported in a large number of environmental contamination studies [79,80,81], and it could also be of interest in a chemical food-safety perspective applied to several veterinary residue families, including protein hormones, such as rGH.

## Figures and Tables

**Table 1:** Endogenous and recombinant bovine growth hormone characteristics (* the bGH shows heterogeneity at position 127 with either a valine or a leucine due to allelic polymorphism. The majority form is Ala-Phe with Leu at position 127 (variant 2 in the Table)) (** from [10,11]).

Name	Number of Amino Acid (Mass)	N-Terminal	126/127 *	pI **
bGH variant 1	191 (21,788 Da)	AFPAMSLSDGLFANAVLR-...	V	7.8–8.2
bGH variant 2	191 (21,802 Da)	AFPAMSLSDGLFANAVLR-…	L	
bGH variant 3	190 (21,717 Da)	FPAMSLSDGLFANAVLR-…	V	
bGH variant 4	190 (21,731 Da)	FPAMSLSDGLFANAVLR-…	L	
rbGH Posilac (Elanco)— Met-rbGH	191 (21,851 Da)	MFPAMSLSDGLFANAVLR-…		7.9
rbGH Boostin (Lg Chem)— Ala-rbGH	191 (21,788 Da)	AFPAMSLSDGLFANAVLR-...	V	8.2

**Table 2:** Bovine milk proteins physico-chemical properties (adapted from * [49] and ** [50]).

Protein Identification	Proportion (% in Protein Fraction of Bovine Milk)	Theoretical Mass (Da) *	pI **
α_s1_-CN B-8P	32.4	23,600.47	4.4–4.8
α_s1_-CN B-9P		23,680.47	
α_s2_-CN A-10P	8.5	25,133.34	
α_s2_-CN A-11P		25,213.34	
α_s2_-CN A-12P		25,293.34	
α_s2_-CN A-13P		25,373.34	
α_s2_-CN A-14P		25,453.34	
β-CN A^1^-5P	26.1	24,008.32	4.8–5.1
β-CN A^2^-5P		23,968.31	
β-CN B-5P		24,077.39	
β-CN I-5P		23,950.36	
κ-CN A-1P	9.4	19,026.54	5.3–5.8
κ-CN A-2P		19,106.54	
κ-CN B-1P		18,994.59	
κ-CN B-1P-G		19,650.82	
κ-CN B-2P		19,074.59	
α-La B	3.6	14,176.8	4.2–4.5
α-La B-G		14,500.9	
β-Lg A	9.8	18,355.45	5.1
β-Lg B		18,269.41	
β-Lg D		18,268.41	
Others (BSA, Lf, Lp, Ig, MFGM)	10.2		

Bovine Serum Albumine (BSA), Lactoferrin (Lf), Lactoperoxidase (Lp), Immunoglobulin (Ig), Milk fat globule membrane (MFGM).

**Table 3:** Indirect analytical strategies in milk to detect rbGH administration.

Strategy	Analytical Technique	Results	Ref.
IGF-1	BIA ELISA	LOQ_BIA_ = 4.0 ng/mL LOQ_ELISA_ = 0.5–1.0 ng/mL	[56]
Antibodies anti-rbGH	FCIA	False compliant = 33%	[57]
	Protein microarray based fluorescence immunoassay	Results in accordance with the FCIA reference method in milk	[65]
Transcriptomics	RT-PCR	4 potential biomarkers: CCND1, IL-1, TNF, IGF-1R	[60]
	RT-PCR	4 biomarkers confirmed	[61]

Insulin-like growth factor I (IGF-I); Recombinant bovine Growth Hormone (rbGH).

**Table 4:** Analytical strategies to monitor rbGH residues in milk.

Strategy	Analytical Strategy				Performances			Ref.
	Sample Preparation	External Standard	Detection	Transitions	Concentration (LOD/LOQ/CCa)	Precision	Recovery	
rbGH residues	SPE C4—MeOH precipitation—Tryptic Digestion	(MFP(A13C)MS(L13C)SG(L13C)F(A13C)N(A13C)V(L13C)R) rbST tryptic N term peptide	LC-MS/MS (QqQ) ESI+	913.2 > 774.1 913.2 > 1047.7	CCα = 1.2 ng/mL	RSD 18%	-	[69]
	Skimming—Immunoprecipitation—Tryptic digestion	(MFP(A13C)MS(L13C)SG(L13C)F(A13C)N(A13C)V(L13C)R) rbST tryptic N term peptide	LC-MS/MS (QqQ) ESI+	913.2 > 774	LOD = 0.07 ng/mL CCα = 0.08 ng/mL	CV > 100%	162%	[70]
	Acetonitrile depletion with slight modifications	-	LC-HRMS (LTQ Orbitrap) ESI+	not specified	-	-	-	[71]
	Skimming—Immunoprecipitation—Tryptic digestion	^13^C_6_ ^15^N_4_-bST tryptic N term peptide	LC-MS/MS (QqQ) ESI+	Met-bGH: 913.1 > 774.1 913.1 > 1047.6 Ala-bGH: 883.1 > 774.1 883.1 > 1047.6 883.1 > 960.6	CCα = 2.3 ng/mL	-	-	[37]

Solid Phase Extraction (SPE), methanol (MeOH), Liquid chromatography tandem mass spectrom-etry (LC-Ms/MS), triple quadrupole (QqQ), Electrospray Ionisation (ESI), Decision limit (CCα); Relative standard deviation (RSD), Limit of detection (LOD), Coefficient of Variation (CV), High resolution mass spectrometry (HRMS).

## Supplementary Materials

The following supporting information can be downloaded at: https://www.mdpi.com/article/10.3390/foods11030274/s1, Table S1: Recombinant growth hormone (rGH) direct methods in several species in blood, Table S2: Recombinant growth hormone (rGH) indirect methods in several species and matrices.
