# Supplementary material for: The Promise and Challenges of Determining Recombinant Bovine Growth Hormone in Milk
Source: Foods. 2022 Jan 20;11(3):274. doi: 10.3390/foods11030274 (PMC8834339; doi:10.3390/foods11030274)
Supplement: Supplementary file 1 [file foods-11-00274-s001.zip › foods-1517040-supplementary.pdf]

SUPPLEMENTARY MATERIAL

Table S1: Recombinant growth hormone (rGH) direct methods in several species in blood.

Table S2: Recombinant growth hormone (rGH) indirect methods in several species and matrices.

SUPPLEMENTARY MATERIAL

Table S1: Recombinant growth hormone (rGH) direct methods in several species in blood.

Strategy	Specie		Matrix	Delivered dose	Analytical Strategy<br>Sample preparation	Analytical Strategy<br>Detection	Limit of detection	Detection window	Method Scope	Ref.
**Total bGH (Endogenous + recombinant bGH)**	Bovine		Plasma	2 Lactatropin (500 mg) injections (2 weeks apart)		RIA	0.5 ng/mL	1 to 14 days	Screening	[1]
	Bovine		Plasma			ELISA	0.2 ng/mL		Screening	[2]
	Bovine		Plasma			BIA	8 ng/mL		Screening	[3]
	Bovine		Plasma			ELISA	66 ng/mL		Screening	[4]
**Recombinant GH**	Bovine		Plasma		Trypsin digestion	HPLC-ESI-MS	1 ng/mL		Confirmation	[5]
	Equine		Plasma	EquiGen-5® (25 µg/kg) for 14 days	Ammonium sulphate precipitation – SPE C4 – Cold methanol precipitation – Trypsin digestion	HPLC-MS/MS	10 ng/mL	1 to 48 hours	Confirmation	[74]
	Caprine		Plasma	2 Lactatropin (500 mg) injections (2 days apart)	Ammonium sulphate precipitation – SPE C4 – Cold methanol precipitation – Trypsin digestion	HPLC-LTQ-Orbitrap	10 ng/mL	1 to 4 days	Confirmation	[7]
	Bovine		Serum	2 Lactatropin (500 mg) injections (2 days apart)	Ammonium sulphate precipitation – SPE C4 – Cold methanol precipitation – Trypsin digestion	HPLC-MS/MS	2.5 ng/mL	Up to 2 weeks after injection	Confirmation	[8]
	Bovine (Buffalo)		Serum	Biweekly injections of Boostin (500 mg) for 12 weeks	Ammonium sulphate precipitation – SPE C4 – Cold methanol precipitation – Trypsin digestion	HPLC-MS/MS	0.25 ng/mL	1 to 9 days of the treatment	Confirmation	[9]
	Bovine		Serum	2 Lactatropin (500 mg) injections (1 w apart)	Immuno-affinity enrichment on monolithic micro-columns – Trypsin digestion	UPLC-MS/MS	0.8 ng/mL	1 to 21 days of the treatment	Confirmation	[10]
	Bovine		Serum	2 Lactatropin (500 mg) injections (2 w apart)	Immuno-affinity enrichment with magnetic beads coated with rbGH specific antibodies – Trypsin digestion	UPLC-MS/MS	0.11 ng/mL	1 to 9 days of the treatment	Confirmation	[119]
	Equine		Plasma	EquiGen-5® daily injections for 14 d	Ammonium sulphate precipitation – SPE C4 – Carbon chloride then Methanol precipitation – Trypsin digestion	UPLC-MS/HRMS	reGH : 0.5 ng/mL rpGH : 1.25 ng/mL		Confirmation	[127]
	Trout		Plasma	1 Lactatropin (500 mg) injection	Ammonium sulphate precipitation – SPE C4 – MeOH precipitation – Trypsin digestion	UPLC-MS/MS	0.5 µg/mL	1 to 30 days	Confirmation	[13]

SUPPLEMENTARY MATERIAL

Table S2: Recombinant growth hormone (rGH) indirect methods in several species and matrices.

Biomarkers monitored		Specie	Matrix	Delivered dose	Target analytes or biomarkers	Analytical Detection	Limit of detection	Detection window	Ref.
**IGF-1**		Bovine	Serum	2 Lactatropin (500 mg) injections (2 weeks apart)	IGF-1	RIA		2-14 days	[1]
		Bovine	Serum		IGF-1	ECLIA	2 pg/mL		[14]
		Bovine	Serum	2 Lactatropin (500 mg) injections (2 weeks apart, twice)	IGF-1 and IGFBP2	FCIA			[15]
		Bovine	Plasma		IGFBP2	ELISA			[16]
		Equine			IGF-1	HPLC-ESI-MS	310 - 1480 ng/mL		[17]
		Equine			IGF-1 (peptides)	HPLC-ESI-MS	30 - 500 ng/mL		[18]
		Equine	Serum	Equigen daily injections (14 days treatment)	IGF-1	HPLC-ESI-MS	318 - 898 ng/mL		[19]
**Anti-rbGH antibodies**		Bovine	Serum	4 Lactatropin (500 mg) injections (2 weeks apart, twice)	Anti-rbGH antibodies	FCIA			[15]
		Bovine	Serum		Anti-rbGH antibodies	ELISA	rbGH-M : 0.05 ng/mL<br>rbGH-LG : 0.10 ng/mL		[20]
		Bovine	Plasma		Anti-rbGH antibodies	ELISA			[16]
		Bovine	Serum	2 Lactatropin (500 mg) injections (3 days apart)	Anti-rbGH antibodies	ELISA		9-23 days	[21]
		Bovine (Buffalo)	Serum	1 Boostin (500 mg) injection every 14 days (for 10 weeks)	Anti-rbGH antibodies	ELISA			[22]
**Omics Field**	Metabolomics	Bovine	Plasma	4 Lactatropin (500 mg) injections (2 weeks apart, twice)	IGF-1, UR, NEFA, INS, COL	Model with 21 blood parameters		1-9 days	[23]
		Equine	Urine	EquiGen-5® (18µg/kg) for 14 days	Glucose, L-carnithine, actylcarnithine, δ-12-prostaglandin-J2	HPLC-HRMS<br>HILIC-HRMS			[24]
	Transcriptomics	Bovine (Buffalos)	White blood cells	1 Boostin (500 mg) injection every 14 days (for 10 weeks)	GADPH, ACTB, 18S, YWHAZ, SDHA, HMBS, SF3A1, EEFIA2	RT-PCR	Candidates not influenced by rbGH treatment		[25]

		Bovine (Buffalos)	Skeletal muscle	1 Boostin (500 mg) injection every 14 days (for 10 weeks)	IGF-1, IGF-2, 6 IGFBPs, IGF-1R, ALS, GHR, GHR 5′-UTR	ELISA	Somatotropic axis genes not affected by rbGH treatment	[26]
		Bovine (Buffalo)	White blood cells	1 Boostin (500 mg) injection every 14 days (for 12 weeks)	AQP3, BIRC5, CdC20, IGHG1, KIF22, LMAN1, LMTK2, MZB1, NUSAP1	RT-PCR	White blood cells genes not useful to detect rbGH treatment	[27]

Références

1. Schams, D.; Graf, F.; Meyer, J.; Graule, B.; Mauthner, M.; Wollny, C. Changes in hormones, metabolites, and milk after treatment with sometribove (recombinant methionyl bST) in Deutsches Fleckvieh and German black and white cows. J. Anim. Sci. 1991, 69, 1583-1592. https://doi.org/10.2527/1991.6941583x.
2. Zwickl, C.M.; Smith, H.W.; Bick, P.H. Rapid and sensitive ELISA method for the determination of bo-vine somatotropin in blood and milk. J. Agric. Food Chem. 1990, 38, 1358-1362. https://doi.org/10.1021/jf00096a013.
3. Heutmekers, T.H.; Bremer, M.G.; Haasnoot, W.; Nielen, M.W. A rapid surface plasmon resonance (SPR) biosensor immunoassay for screening of somatotropins in injection preparations. Anal. Chim. Acta 2007, 586, 239-245. https://doi.org/10.1016/j.aca.2006.11.047.
4. Suárez-Pantaleón, C.; Huet, A.; Kavanagh, O.; Lei, H.; Dervilly-Pinel, G.; Le Bizec, B.; Situ, C.; Delahaut, P. Production of polyclonal antibodies directed to recombinant methionyl bovine somatotropin. Anal. Chim. Acta 2013, 761, 186-193. https://doi.org/10.1016/j.aca.2012.11.041.
5. Rochut, N.; Le Bizec, B.; Monteau, F.; Andre, F. ESI-MS for the measurement of bovine and porcine somatotropins. Analusis 2000, 28, 280-284. https://doi.org/10.1051/analusis:2000280280.
6. Bailly-Chouriberry, L.; Pinel, G.; Garcia, P.; Popot, M.-A.; LE Bizec, B.; Bonnaire, Y. Identification of Recombinant Equine Growth Hormone in Horse Plasma by LC?MS/MS: A Confirmatory Analysis in Doping Control. Anal. Chem. 2008, 80, 8340-8347. https://doi.org/10.1021/ac801234f.
7. Le Breton, M.-H.; Rochereau-Roulet, S.; Pinel, G.; Bailly-Chouriberry, L.; Rychen, G.; Jurjanz, S.; Gold-mann, T.; LE Bizec, B. Direct determination of recombinant bovine somatotropin in plasma from a treated goat by liquid chromatography/high-resolution mass spectrometry. Rapid Commun. Mass Spec-trom. 2008, 22, 3130-3136. https://doi.org/10.1002/rcm.3712.
8. Le Breton, M.-H.; Rochereau-Roulet, S.; Che?reau, S.; Pinel, G.; Delatour, T.; LE Bizec, B. Identification of Cows Treated with Recombinant Bovine Somatotropin. J. Agric. Food Chem. 2009, 58, 729-733. https://doi.org/10.1021/jf903032q.
9. Castigliego, L.; Armani, A.; Grifoni, G.; Mazzi, M.; Boselli, C.; Guidi, A.; Donzelli, R.; Saba, A. A LC-MS-MS method to detect recombinant bovine somatotropin misuse in buffalos. Anal. Bioanal. Chem. 2016, 408, 4917-4926. https://doi.org/10.1007/s00216-016-9578-9.
10. Smits, N.G.E.; Blokland, M.; Wubs, K.L.; Nessen, M.A.; Van Ginkel, L.A.; Nielen, M.W.F. Monolith im-muno-affinity enrichment liquid chromatography tandem mass spectrometry for quantitative protein analysis of recombinant bovine somatotropin in serum. Anal. Bioanal. Chem. 2015, 407, 6041-6050. https://doi.org/10.1007/s00216-015-8775-2.
11. Robert, C.; Huet, A.-C.; Suárez-Pantaleón, C.; Brasseur, A.; Delahaut, P.; Gillard, N. Development of a confirmatory method for detecting recombinant bovine somatotropin in plasma by immunomagnetic precipitation followed by ultra-high performance liquid chromatography coupled to tandem mass spectrometry. Food Addit. Contam. Part A 2017, 34, 1925-1934. https://doi.org/10.1080/19440049.2017.1364429.
12. Wong, K.-S.; Chan, G.H.M.; Ho, E.N.; Wan, T.S. Simultaneous detection of recombinant growth hor-mones in equine plasma by liquid chromatography/high-resolution tandem mass spectrometry for doping control. J. Chromatogr. A 2016, 1478, 35-42. https://doi.org/10.1016/j.chroma.2016.11.032.
13. Rochereau-Roulet, S.; Gicquiau, A.; Morvan, M.L.; Blanc, G.; Dervilly-Pinel, G.; LE Bizec, B. Recombinant bovine growth hormone identification and the kinetic of elimination in rainbow trout treated by LC-MS/MS. Food Addit. Contam. Part A 2013, 30, 1020-1026. https://doi.org/10.1080/19440049.2013.787650.
14. McGrath, M.F.; Bogosian, G.; Fabellar, A.C.; Staub, R.L.; Vicini, J.L.; Widger, L.A. Measurement of Bo-vine Somatotropin (bST) and Insulin-like Growth Factor-1 (IGF-1) in Bovine Milk Using an Electro-chemiluminescent Assay. J. Agric. Food Chem. 2008, 56, 7044-7048. https://doi.org/10.1021/jf800696d.
15. Smits, N.G.E.; Bremer, M.G.E.G.; Ludwig, S.K.J.; Nielen, M.W.F. Development of a flow cytometric im-munoassay for recombinant bovine somatotropin-induced antibodies in serum of dairy cows. Drug Test. Anal. 2011, 4, 362-367. https://doi.org/10.1002/dta.336.
16. Scippo, M.; Degand, G.; Duyckaerts, A. Identification of bovine somatotropine-treated cows. Ann. Med. Vet. 1997, 141, 381-390.
17. Bobin, S.; Popot, M.A.; Bonnaire, Y.; Tabet, J.C. Approach to the determination of insu-lin-like-growth-factor-I (IGF-I) concentration in plasma by high-performance liquid chromatog-raphy-ion trap mass spectrometry: Use of a deconvolution algorithm for the quantification of multi-protonated molecules in electrospray ionization. Analyst 2001, 126, 1996-2001. https://doi.org/10.1039/b105022m.

18. de Kock, S.S.; Rodgers, J.P.; Swanepoel, B.C. Growth hormone abuse in the horse: Preliminary assess-ment of a mass spectrometric procedure for IGF-1 identification and quantitation. Rapid Commun. Mass Spectrom. 2001, 15, 1191-1197. https://doi.org/10.1002/rcm.363.
19. Popot, M.-A.; Woolfitt, A.R.; Garcia, P.; Tabet, J.-C. Determination of IGF-I in horse plasma by LC electrospray ionisation mass spectrometry. Anal. Bioanal. Chem. 2008, 390, 1843-1852. https://doi.org/10.1007/s00216-008-1889-z.
20. Castigliego, L.; Iannone, G.; Grifoni, G.; Rosati, R.; Gianfaldoni, D.; Guidi, A. Natural and recombinant bovine somatotropin: Immunodetection with a sandwich ELISA. J. Dairy Res. 2006, 74, 79-85. https://doi.org/10.1017/s0022029906002159.
21. Rochereau-Roulet, S.; Gaudin, I.; Chéreau, S.; Prévost, S.; André-Fontaine, G.; Pinel, G.; Le Bizec, B. Development and validation of an enzyme-linked immunosorbent assay for the detection of circulating antibodies raised against growth hormone as a consequence of rbST treatment in cows. Anal. Chim. Acta 2011, 700, 189-193. https://doi.org/10.1016/j.aca.2011.01.035.
22. Castigliego, L.; Tinacci, L.; Armani, A.; Boselli, C.; Grifoni, G.; Mazzi, M.; Guidi, A. Serum responsive-ness to recombinant bovine somatotropin in buffalo: A three-month lactation study using an ac-id-stripping ELISA for screening. Drug Test. Anal. 2016, 9, 646-656. https://doi.org/10.1002/dta.1994.
23. Doué, M.; Dervilly-Pinel, G.; Cesbron, N.; Stefani, A.; Moro, L.; Biancotto, G.; LE Bizec, B. Clinical biochemical and hormonal profiling in plasma: A promising strategy to predict growth hormone abuse in cattle. Anal. Bioanal. Chem. 2015, 407, 4343-4349. https://doi.org/10.1007/s00216-015-8548-y.
24. Boyard-Kieken, F.; Dervilly-Pinel, G.; Garcia, P.; Paris, A.-C.; Popot, M.-A.; LE Bizec, B.; Bonnaire, Y. Comparison of different liquid chromatography stationary phases in LC-HRMS metabolomics for the detection of recombinant growth hormone doping control. J. Sep. Sci. 2011, 34, 3493-3501. https://doi.org/10.1002/jssc.201100223.
25. Castigliego, L.; Armani, A.; Li, X.; Grifoni, G.; Gianfaldoni, D.; Guidi, A. Selecting reference genes in the white blood cells of buffalos treated with recombinant growth hormone. Anal. Biochem. 2010, 403, 120-122. https://doi.org/10.1016/j.ab.2010.04.001.
26. Castigliego, L.; Armani, A.; Grifoni, G.; Rosati, R.; Mazzi, M.; Gianfaldoni, D.; Guidi, A. Effects of growth hormone treatment on the expression of somatotropic axis genes in the skeletal muscle of lac-tating Holstein cows. Domest. Anim. Endocrinol. 2010, 39, 40-53. https://doi.org/10.1016/j.domaniend.2010.02.001.
27. Castigliego, L.; Carrieri, F.; Armani, A.; Mazzi, M.; Boselli, C.; Grifoni, G.; Gianfaldoni, D.; Guidi, A. Selection of Biomarkers from Differentially Expressed Genes in Leukocytes of Buffalos Treated with Recombinant Bovine Somatotropin: The Importance of Sample Size for Reliable Discriminating Sys-tems. J. Buffalo Sci. 2016, 5, 1-13. https://doi.org/10.6000/1927-520x.2016.05.01.
